# Repertoire of noncoding RNAs in corpus luteum of early pregnancy in buffalo (*Bubalus bubalis*)

**DOI:** 10.14202/vetworld.2017.1129-1134

**Published:** 2017-09-25

**Authors:** A. Jerome, S. M. K. Thirumaran, S. N. Kala

**Affiliations:** 1 ICAR-Central Institute for Research on Buffaloes, Hisar - 125 001, Haryana, India; 2 ICAR-Central Sheep and Wool Research Institute, Mannavanur, Tamil Nadu, India

**Keywords:** buffalo, corpus luteum, noncoding RNA, pregnancy

## Abstract

**Aim:**

The present study was designed to identify other noncoding RNAs (ncRNAs) in the corpus luteum (CL) during early pregnancy in buffalo.

**Materials and Methods:**

For this study, CL (n=2) from two buffalo gravid uteri, obtained from the slaughter house, was transported to laboratory after snap freezing in liquid nitrogen (−196°C). The stage of pregnancy was determined by measuring the crown-rump region of the fetus. This was followed by isolation of RNA and deep sequencing. Post-deep sequencing, the obtained reads were checked and aligned against various ncRNA databases (GtRNA, RFAM, and deep guide). Various parameters, namely, frequency of specific ncRNAs, length, mismatch, and genomic location target in several model species were deciphered.

**Results:**

Frequency of piwi-interacting RNAs (piwi-RNAs), having target location in rodents and human genomes, were significantly higher compared to other piwi-RNAs and ncRNAs. Ribosomal RNAs (rRNAs) deduced had nucleotides (nts) ranging from 17 to 50 nts, but the occurrence of small length rRNAs was more than lengthier fragments. The target on 16S rRNA species confirms the conservation of 16S rRNA across species. With respect to transfer RNA (tRNA), the abundantly occurring tRNAs were unique with no duplication. Small nucleolar RNAs (snoRNAs), identified in this study, showed a strong tendency for coding box C/D snoRNAs in comparison to H/ACA snoRNAs. Regulatory and evolutionary implications of these identified ncRNAs are yet to be delineated in many species, including buffaloes.

**Conclusion:**

This is the first report of identification of other ncRNAs in CL of early pregnancy in buffalo.

## Introduction

Corpus luteum (CL), a dynamic ovarian endocrine structure, through its sequential morphological and functional changes, helps in the production and secretion of progesterone. This release of progesterone is imperative for controlling estrus cycle along with its priming effect for estrus behavior and maintenance of pregnancy [1]. The synchronized functioning of CL is controlled by many paracrine, endocrine factors and genes during the estrous cycle and pregnancy [2]. Embryonic mortality in buffaloes has been reported to occur between day 25 and 40 of pregnancy, making it a critical period during pregnancy [3]. Recently, noncoding RNAs (ncRNAs) have been identified in varied biological tissues, including CL [4]. MicroRNAs (miRNAs) are noncoding single-strand RNAs, 17-22 nts in length, that play a role in the regulation of gene expression post-transcriptionally either by cleavage of target mRNA, deadenylation of target mRNA, and/or inhibition of translation [5]. The role of miRNA in reproduction has been documented by several studies [4-8], but studies reporting other ncRNAs are lacking or sparse in buffaloes. In recent times, studies have deciphered ncRNAs, namely, piwi-RNA, snoRNA, tRNA, and rRNA in several model species such as fruit fly, rodents, nematode, bats, and humans [4-8].

In this regard, there is increasing evidence that these ncRNAs are playing a predominant role in the various cellular mechanisms. Piwi-RNA is more specifically restricted to germline playing a regulatory role in epigenetics and transposition [8]. Similarly, snoRNAs give rise to conserved proteins forming defined C/D and H/ACA small nucleolar RNA-protein complexes (snoRNPs). They are proposed to be involved in alteration and cleavage of nascent rRNA transcripts in various species. Likewise, other RNAs, namely, transfer RNAs (tRNAs) and ribosomal RNAs (rRNAs) have role in 2’-O-ribose methylation and pseudouridylation of specific rRNA nts, ribosomal processing, and protein synthesis [9]. Although the biogenesis of ncRNAs is not fully understood, their presence has implication in various biological processes. These ncRNAs target various critical genes for supporting luteal progesterone production during pregnancy [10].

Although such studies throw light on miRNAs, there is lack of identification of other ncRNAs in CL of early pregnancy in buffaloes. Thus, the present study was aimed to identify other ncRNAs (piwi-RNA, snoRNA, tRNA, and rRNA) in the CL of early pregnancy in buffalo by deep sequencing.

## Materials and Methods

### Ethical approval

This study was approved by Institute Animal Ethical Committee.

### Sampling

For this study, CL (n=2) from the gravid uteri of two buffaloes, from slaughter house, were collected and snap frozen in liquid nitrogen (−196°C) before transporting them to laboratory, the stage of pregnancy was determined by measuring the crown-rump region of the fetus and calculated using the calculator (fetal age calculator; http://www.ansci.wisc.edu/jjp1/ansci_repro/lab/female_anatomy/crown_rump_calculators.htm).

### Isolation of RNA, library preparation and sequencing

Isolation of RNA was carried out following kit’s protocol (RNA Easy Kit, Qiagen, USA), from 30 mg of CL tissue from each buffalo. RNA purity, concentration, and integrity number (RIN) of the samples was checked using Agilent 2100 Bioanalyzer (Agilent Technologies, USA). Samples having RIN value >8 were used for further processing. The isolated RNA (1 µg) was used for small RNA library preparation according to manufacturer’s protocol (Sample preps truseq/truseqsmallrna, Illumina, USA) and single-end sequencing was performed using the standard protocol in Illumina HiSeq 2000 sequencer (Scigenom Pvt., Ltd., India).

### Quality checking of reads and bioinformatics analyses

Post-deep sequencing, the obtained reads were checked for base quality score distribution, sequence quality score distribution, average base content per read, and GC distribution in the reads before further processing. The reads possessing GC content more than 50 and Phred score >30 were used for further *in silico* analysis. The 5’ and 3’ adapters’ sequences were removed using cutadapt tool (version 1.3) [11] from the raw reads. The adapters were 5’ RNA Adapter: 5’ GTTCAGAGTTCTACAGTCCGACGATC and 3’ RNA Adapter: 5’ TGGAATTCTCGGGTGCCAAGG. The base “U” from adapter was replaced as “T” while adapter trimming process.

The obtained adaptor removed reads were aligned against mirBASE database and followed by ncRNA sequence databases using Bowtie program (version 0.12.9) [12]. Following this, read sequences not aligning with mirBASE database [13,14] were removed from the adapters trimmed reads (read length with >16 bp). Adapter trimmed reads not matching to mirBASE database were aligned against various ncRNA databases, namely, GtRNA (http://gtrnadb.ucsc.edu/) [15], piwi-RNA (http://pirnabank.ibab.ac.in/) [16], RFAM (http://rfam.xfam.org/) [17], deep guide (http://deepbase.sysu.edu.cn/prediction.php) [18], and snoRNA (http://lowelab.ucsc.edu/snoRNAdb/) [19]. Reads lengths varying at both 3’ and 5’ and possessing a single or no mismatch inside of the sequence were allowed in the alignment. Various parameters, namely, frequency of specific ncRNAs, length, mismatch, and genomic location target in several model species were deciphered. Using these strategies, *in silico* mining of other ncRNAs in the CL of pregnancy in buffaloes were carried out.

## Results

**Figure-1:**
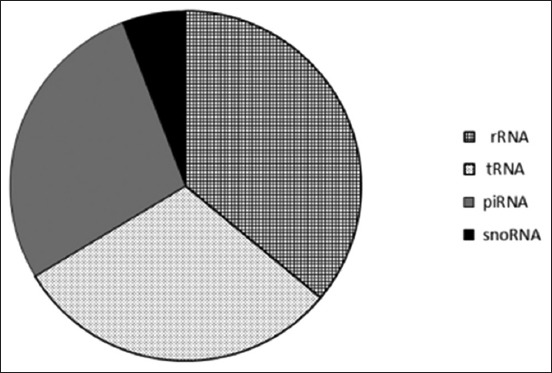
Proportion of other noncoding RNAs excluding microRNAs identified in corpus luteum of early pregnancy in buffaloes.

From the measured crown-rump distance of the fetus, the stage of pregnancy was calculated to be approximately 30-35 days. The total amount of RNA obtained from each sample was approximately 1 µg with 260/280 ratio of 2.04 and RIN value of the samples was >8. About 98.03% of total data passed >30 Phred score (Table-1). A total number of reads aligning and not aligning to tRNA, rRNA, piwi-RNA, and snoRNA database were 173230 (5.15%) and 3679491 (94.85%), respectively, and their percent is shown in Table-2 and Figure-1. rRNA, tRNAs, piwi-RNA, and snoRNA occurring at high frequency along with their targets are shown in Tables-3-6.

**Table-1:** Summary of raw reads quality post-sequencing.

Sample	Mean read quality (Phred score)	%Q<10	%Q 10-20	%Q 20-30	%Q>30	Number of bases	%GC
A	38.88	0.28	0.26	0.96	98.51	1527.8	53.65
B	38.46	0.33	0.27	1.37	98.03	1319.93	56.98

**Table-2:** Reads aligned to specific ncRNAs databases.

Read parameter	Read count (%)
Number of reads aligned to rRNA databases	62415 (1.854)
Number of reads aligned to tRNA databases	52592 (1.562)
Number of reads aligned to piwi-RNA databases	48129 (1.429)
Number of reads aligned to snoRNA databases	10094 (0.299)

ncRNAs=Noncoding RNAs, rRNA=Ribosomal RNA, tRNA=Transfer RNA, piwi-RNA=piwi-interacting RNA, snoRNA=Small nucleolar RNA

**Table-3:** List of piwi-RNA in the CL of early pregnancy in buffalo.

Sequence	Sequence frequency in sample	Length bp	Total matches with target	Target_ID (strand: Mismatch)
CACCAAGAGGCTGTACC	136770	17	1	mmu_piR_014372|gb|DQ692529|Mus_musculus: 7:125123491:125123522:Minus(-:1)
TATTGCACTTGTCCCGGCCTGT	109390	22	1	dme_piR_000201|Drosophila_melanogaster: 3R: 21472282:21472303:Plus(+:1)
CACCAAGAGGCTGTACCA	41608	18	1	mmu_piR_014372|gb|DQ692529|Mus_musculus: 7:125123491:125123522:Minus(-:1)
TGAGGTAGTAGGTTGTATAGT	35671	21	1	hsa_piR_008113|gb|DQ581032|Homo sapiens(+:0)
ACCGGGTGCTGTAGGCTT	25087	18	1	hsa_piR_016735|gb|DQ593039|Homo sapiens (+:0)
TGAGGTAGTAGGTTGTATA	20829	19	1	hsa_piR_008113|gb|DQ581032|Homo sapiens (+:0)
GGCCGTGATCGTATAGTGGTTAGTACTCT	12361	29	1	hsa_piR_020365|gb|DQ597975|Homo sapiens (+:0)
TATTGCACTTGTCCCGGCCT	11487	20	1	dme_piR_000201|Drosophila_melanogaster: 3R: 21472282:21472303:Plus(+:1)
TGAGGTAGTAGATTGTATAGT	10344	21	1	hsa_piR_008112|gb|DQ581031|Homo sapiens (+:0)
TTTCGAGGCCCTGTAAT	10090	17	1	dr_piR_0055211|Danio_rerio: 20:3365130:3365155:Plus(-:1)
ATACCGGGTGCTGTAGGCTT	9789	20	1	hsa_piR_016735|gb|DQ593039|Homo sapiens (+:0)
TGAGGTAGTAGATTGTATA	9542	19	1	hsa_piR_008112|gb|DQ581031|Homo sapiens (+:0)
ACCGGGTGCTGTAGGCTTT	9468	19	1	hsa_piR_016735|gb|DQ593039|Homo sapiens (+:1)
CTTTCGAGGCCCTGTAAT	7476	18	1	dr_piR_0055211|Danio_rerio: 20:3365130:3365155:Plus(-:1)
CTGGACGCGAGCCGGGCCCTTC	6723	22	1	dr_piR_0023467|Danio_rerio: 7:11864:11891:Plus(+:1)

Piwi-RNA=Piwi-interacting RNA, CL=Corpus luteum

**Table-4:** List of rRNA in the CL of early pregnancy in buffalo.

Sequence	Sequence frequency in sample	Length in bp	Total matches with target	Target ID (strand: Mismatch)
GTTCGCGCTTTCCCCTG	38979	17	1	Unidentified bacterium partial 16S rRNA gene/AJ518718.1/4-355 (-:1)
CGTTCGCGCTTTCCCCTG	33368	18	1	Mytilimeria nutallii 18S ribosomal RNA gene/AY192704.1/1-662 (-:1)
ATACTCTCCTTGGTGGCC	12887	18	1	Uncultured bacterium TIHP368-03 gene for 16S rRNA/AB031629.1/2-495 (-:1)
TACTCTCCTTGGTGGCCA	8511	18	1	*Syntrophus* sp. 16S rRNA gene/AJ133795.1/16-562 (-:1)
TACTCTCCTTGGTGGCC	7366	17	1	Uncultured bacterium TIHP368-03 gene for 16S rRNA/AB031629.1/2-495 (-:1)
CCCGGGGCCGGGAGCGGCC	5556	19	1	Mouse RNase P RNA subunit gene/L08802.1/5-290 (-:1)
CCGCGCGGCTCCCTCCC	4387	17	1	Uncultured gamma proteobacterium partial 16S rRNA gene/Z77472.1/1-302 (-:1)
CCCGCGGGGGCCCGGGCACCC	3302	21	1	*Leishmania tarentolae* tRNA-Asp gene/AF409072.1/1-72 (+:1)
CTCCACGCCCGGGGCCG	2967	17	1	*Prevotella micans* strain E7.56 16S rRNA gene/AC097484.3/133832-133932 (-:1)
TGCGTTCGCGCTTTCCCCTG	2508	20	1	*Desulfovibrio vulgaris* subsp. vulgaris str./AE017309.2/30047-30119 (-:1)
CCTCCACGCCCGGGGCCG	2448	18	1	Uncultured bacterium clone unel4 16S rRNA gene/AY186798.1/3-495 (-:1)
CGGGGCCGCCCCCGCGGGCC	2152	20	1	*Senecio cambrensis* partial 18S rRNA gene/X59119.1/1-533 (-:1)
CCACCACGTTCCCGTGG	2010	17	1	Uncultured planctomycete clone18 16S rRNA gene/AF271314.1/3-495 (-:1)

rRNA=Ribosomal RNA, CL=Corpus luteum

**Table-5:** List of tRNA in the CL of early pregnancy in buffalo.

Sequence	Sequence frequency in sample	Length in bp	Total matches with target	Target ID (strand: Mismatch)
GCCCGGCTAGCTCAGTCGGTAGAGCATGAGACTC	556,095	34	1	Gorilla_gorilla_gorilla_chr6.trna13-LysCTT (+:0)
GCATTGGTGGTTCAGTGGTAGAATTCTCGCCTG	349,841	33	1	Danio_rerio_chr5.trna803-GlyGCC (+:0)
TCCCTGGTGGTCTAGTGGTTAGGATTCGGCGCTC	333,056	34	1	Danio_rerio_chr22.trna444-GluCTC (+:0)
GTTTCCGTAGTGTAGTGGTTATCACGTTCGCCT	224,679	33	1	Gorilla_gorilla_gorilla_chr5.trna19-ValCAC (+:0)
GTTTCCGTAGTGTAGTGGTTATCACGTTCGCCTC	126,835	34	1	Ovis_aries_chr5.trna1170-ValCAC (+:0)
GCATTGGTGGTTCAGTGGTAGAATTCTCGCCT	114,940	32	1	Danio_rerio_chr4.trna5936-GlyGCC (+:0)
GCATGGGTGGTTCAGTGGTAGAATTCTCGCCTG	88,073	33	1	Rattus_norvegicus_chr13_random.trna7-GlyGCC (+:0)
GCATTGGTGGTTCAGTGGTAGAATTCTCGCCTC	80,078	33	1	Danio_rerio_Zv8_NA538.trna46-GlyCCC (+:0)
TCCCTGGTGGTCTAGTGGTTAGGATTCGGCGCTCTC	69,277	36	1	Oryctolagus_cuniculus_chr13.trna668-GluCTC (+:0)
GCATTGGTGGTTCAGTGGTAGAATTCTCGCC	64,801	31	1	Danio_rerio_chr4.trna3778-GlyGCC (+:0)
TCCCTGGTGGTCTAGTGGTTAGGATTCGGCGCT	63,803	33	1	Danio_rerio_chr4.trna4462-GluCTC (+:0)
TCCCTGTGGTCTAGTGGTTAGGATTCGGCGCT	58,788	32	1	Ovis_aries_chr21.trna409-GluTTC (+:1)
TCCCTGGTGGTCTAGTGGTTAGGATTCGGCGCTCT	52,003	35	1	Danio_rerio_chr4.trna7946-GluCTC (+:0)
GCATTGTGGTTCAGTGGTAGAATTCTCGCCTG	48,152	32	1	Loxodonta_africana_scaffold_140.trna3-GluTTC (+:1)
GCGCCGCTGGTGTAGTGGTATCATGCAAGATTCCC	46,461	35	1	Canis_familiaris_chr6.trna1052-GlyCCC (+0:0)

tRNA=transfer RNA, CL=Corpus luteum

**Table-6:** List of snoRNA in the CL of early pregnancy in buffalo.

Sequence	Sequence frequency in sample	Length in bp	Total matches with target	Target-ID (strand: Mismatch)
TCCTGTACTGAGCTGCCCCGAG	4152	22	1	deepBase_guideACA173(-:0)
GTACATGATGACAACTGGCTCCCTCTACTGAAC	3901	33	1	deepBase_guideCD177(+:0)
TCCTGTACTGAGCTGCCCCGA	3648	21	1	deepBase_guideACA173(-:0)
ACCCCGTGATGGAACTGAGGATCTGAGG	2750	28	1	deepBase_guideCD252(+:1)
ATCTGTGATGACTTACA	2518	17	1	deepBase_guideCD72(+:1)
GCGGGTGATGCGAACTGGAGTCTGAGT	2230	27	1	deepBase_guideCD161(+:1)
TCCTGTACTGAGCTGCCCCGAGT	1921	23	1	deepBase_guideACA173(-:1)
CCTTCCTTGGATGTCTGAGTGAC	1678	23	1	deepBase_guideCD143(+:0)
TTCCTTGGATGTCTGAGTGAC	1039	21	1	deepBase_guideCD143(+:0)
CCCCGTGATGGAACTGAGGATCTGAGG	998	27	1	deepBase_guideCD252(+:1)
TGTACATGATGACAACTGGCTCCCTCTACTGAAC	862	34	1	deepBase_guideCD177(+:0)
AATGAAAAGACATGAACACCTGAGA	850	25	1	deepBase_guideCD70(+:1)
TGCTGACGCGGGTGATGCGAACTGGAGTCTGAGT	827	34	1	deepBase_guideCD161(+:1)
TTCCTTGGATGTCTGAGCGAT	792	21	1	deepBase_guideCD39(+:1)
CGCGGGTGATGCGAACTGGAGTCTGAGT	790	28	1	deepBase_guideCD161(+:1)

CL=Corpus luteum, snoRNA=Small nucleolar RNA

## Discussion

This is the first study on profiling ncRNAs in CL of early pregnancy in buffalo. In addition to well-studied ncRNAs, such as snoRNAs and piwi-RNAs, other classes of ncRNA, namely, tRNA and rRNA were deciphered in this study. Piwi-RNAs are small RNA molecules consisting of 24-31 nts and interact with piwi subfamily of proteins. Some of the important regulatory roles of these piwi-RNAs are the germline stem cell maintenance and transposition. In this study, identification of piwi-RNAs from the CL of early pregnancy confirms its direct regulation in germline which is in accordance with earlier reports [20,21]. In species such as rodents and pigs, piwi-RNAs are identified in the testis were confirmed to play role during spermatogenesis. Similarly, piwi-RNAs identified in CL in this study can be implicated in ovarian biology. However, the established role of these piwi-RNAs in transposon silencing, in CL of early pregnancy buffalo is still to be elucidated. Identification of piwi-RNA in ovarian tissue has been documented in neonatal and adult pig ovaries with neonatal ovaries showing abundance of piwi-RNAs as compared to adult ovaries [10]. Such studies are still warranted in buffaloes as biological role of piwi-RNA in mammalian germ cells remains unknown. The target location of abundantly occurring piwi-RNA identified in this study was found in rodents and humans as compared to other piwi-RNAs (Table-3). Studies on regulatory mechanism of piwi-RNAs are still at the nascent stages. In comparison to other ncRNAs, piwi-RNA percentage was found to be higher in comparison to snoRNAs, being in agreement with earlier report [8]. *In silico* analysis has located the piwi-RNAs as intergenic, intronic, and exonic piwi-RNAs, but this was beyond the scope of this study due to lack of genomic annotation of buffalo species. Furthermore, majority of piwi-RNAs sequences deduced consisted of 24-31 nts confirming that piwi-RNAs are not processed by dicer [22].

In addition, reports have shown that piwi-RNA shows a strong bias toward nts preferring uracil at their 5’ ends and adenine residue at position 10 [23,24]. This was not evident or still need to be deciphered from the obtained data in the future. Apart from piwi-RNAs, other ncRNAs deciphered were rRNA, tRNA, and snoRNAs. rRNAs deduced had nts ranging from 17 nts to 50 nts, but the occurrence of small length rRNAs was higher as compared to lengthier fragment. The abundant rRNA targeted the 16S and18S rRNA of various bacteria and eukaryotic organism, respectively (Table-4). Interestingly, the target on 16S rRNA species confirms their conservation and similarity across species which might be due to the presence of 2 mitochondrial (12S and 16S) rRNA molecules in mammalian cells, thereby justifying their targets. In eukaryotic cells, ribosome biogenesis is facilitated by coordinated activity of all three RNA polymerases and associated ribosome assembly factors. Although various factors in ribosome synthesis have been deciphered, understanding their roles is still a daunting task. It is established that rRNA is conserved across archaeal and eukaryotic ribosomes, thereby signifying the commonness in the mechanism of tRNA cognating during decoding [25,26]. Pertaining to tRNA, the abundantly occurring tRNAs were unique with no duplication having more than 30 nts. However, other tRNAs had 17-50 nts (Table-5). Nonetheless, their frequency of occurrence was minimal. It was interesting to note that the abundant tRNAs deduced in this study aligned mostly with the genome of mammals as compared to lower species hinting the conservation of tRNAs genes across mammals. It was noteworthy to deduce that tRNA deduced had their targets in higher species as compared to rRNA on lower forms (bacteria, protozoa, and fungi). The main function of tRNA is aiding the binding sites of aminoacyl-transferRNA (tRNA) by rRNA [27]. It is established that tRNAs consist of secondary structure made of three hairpin loops and a terminal helical stem folding into an L-shaped tertiary structure.

In this study, the frequency of occurrence of lysine, glycine, glutamate, and valine tRNA was higher. The functional role of these specific amino acids in CL tropism of early pregnancy in buffaloes is yet to be delineated. Finally, snoRNAs have been reported in several eukaryotic genomes, namely, plant, drosophila, trypanosomatids, and humans [28]. snoRNAs consists of more than 80 nts necessary for rRNA maturation. In general, snoRNAs consists of two evolutionary conserved sequence elements, namely, box C/D for 2’-O-methylation of the ribose and box H/ACA snoRNAs for snoRNAs guided conversion of uridine nts to pseudouridine. Each class of snoRNAs possesses a characteristic secondary structure and interacts with either C/D or H/ACA box to form highly conserved proteins, that is, snoRNPs. In contrast, highly variable features of snoRNAs in different eukaryotes are their genomic location and mode of transcription. In this study, the identified snoRNAs had their targets on the genomes of model species [28-30]. Furthermore, in eukaryotes, snoRNAs show a strong tendency for box C/D snoRNAs with very few snoRNA genes code for box H/ACA snoRNAs (Table-6) [29] which was in accordance with our study. In most mammals including human, majority of snoRNA genes are intronic, but information are still to be elucidated in buffaloes. The regulatory, evolutionary implications and expression pattern of these ncRNA are yet to be studied in many species, including buffaloes. Although this study has been carried out in small sample size, its importance can be justified in non-model species to cut down the enormous cost of next generation sequencing. Furthermore, smaller studies can provide quicker results for designing larger studies [31]. In summary, the frequencies of piwi-RNA on human and rodent genome were higher as compared to other piwi-RNAs and ncRNAs. The occurrence of smaller rRNA was deduced possessing targets on 16S and 18S rRNA species across species. Transfer RNAs were unique with no duplication and snoRNAs identified in this study showed a strong tendency for coding C/D snoRNAs in comparison to box H/ACA. In conclusion, this study reports the identification of other ncRNAs in CL of early pregnancy in buffalo.

## Authors’ Contributions

JA, SMKT, and SNK: Contributed in study design, collection of samples, and data analysis. JA: Contributed in drafting and revision of manuscript. All authors read and approved the final manuscript.
